# Dual polarized engineering the extinction cross-section of a dielectric wire using graphene-based oligomers

**DOI:** 10.1038/s41598-021-87145-7

**Published:** 2021-04-06

**Authors:** Shiva Hayati Raad, Zahra Atlasbaf

**Affiliations:** Department of Electrical and Computer Engineering, Tarbiat Modares University, Tehran, Iran

**Keywords:** Optics and photonics, Optical materials and structures

## Abstract

In this paper, graphene-coated spherical nanoparticles are arranged around an infinite length dielectric cylinder to enhance its extinction cross-section. Initially, a single longitudinal one-dimensional periodic array is considered in different loci concerning the transverse electric (TE) incident plane wave. It is observed that regardless of the position of the particles, the extinction cross-section of the dielectric cylinder is considerably enhanced with respect to the bare one. Later, by increasing the number of longitudinal plasmonic arrays around the cylinder, each residing in a different azimuthal direction, the extinction cross-section is further manipulated to observe double pronounced Fano resonances. The origin of the Fano resonances is described by considering their planar counterparts constructed by the periodic assembly of plasmonic oligomers. Finally, the hexamer configuration is considered as the prototype, and the effect of various optical, geometrical, and material parameters on the optical response is investigated in detail. Interestingly, due to the spherical symmetry of the cells, the extinction cross-section is also enhanced for the transverse magnetic (TM) incident wave, which is unattainable using a continuous plasmonic cover made of metal or graphene. The potential application of our proposed structure is in the design of reconfigurable conformal optical absorbers and sensors.

## Introduction

Scattering analysis of cylindrical objects covered by patterned metallic elements or plasmonic metals has been the subject of many pieces of research because of the wide range of applications that can be provided by them. For instance, an infinite length dielectric cylinder coated with a sub-wavelength conformal array of slotted Jerusalem crosses can be used as a mantle cloak for both TE and TM polarizations of the incident waves^1^. Also, core–shell cylindrical particles made of noble metals can be used to enhance luminescence, nonlinear optical activities, and for deep sub-wavelength-scale optical wave-guiding^2^. Moreover, a triple-layered tube consisting double metallic and single dielectric layers is proposed as a super-scatterer, designed using the dispersion engineering method^3^. By further increase of the stacked metal-dielectric layers, multiple super-scattering and invisible cloaking states can be observed for the possible application in electromagnetic tagging^4^. For the metal-based cylindrical scatterers, the plasmonic effects are mostly observed for TE polarized wave fields with respect to the cylinder axis^5^.

By the emergence of two-dimensional (2D) graphene material, the aforementioned applications are implemented with the graphene plasmons due to their exotic properties. For instance, graphene patches are wrapped around a cylindrical object to attain an invisible cloak^6^. Moreover, reconfigurable plasmonic waveguides and refractive index sensors are realized using localized surface plasmons of graphene shells around cylindrical objects^7,8^. As another application, double graphene shells around the dielectric cylinders are used to design single and double band super-scatters^9,10^. By attaining multiple peaks and dips in the scattering response using multiple plasmonic layers, an optical device for simultaneous super-scattering and super-cloaking is proposed^11^. Moreover, flatten conformal hyperbolic metamaterials are presented using graphene strips around a cylinder under magnetic bias^12^. The main limitation in all above-mentioned plasmonic devices is that they function only under TE polarized waves^13^. The goal of this paper is to introduce a dual-polarized graphene-based plasmonic cover for cylindrical wires.

Optical covers can be constructed by a periodic arrangement of plasmonic nanoparticles rather than using the metallic sheets with patterned elements which suffer from the practical realizably at high frequencies^14^. By tailoring the corresponding intrinsic surface resistance, optical-resistive sheets and Salisbury absorbers have been implemented^15^. Moreover, by properly adjusting the homogenized surface reactance of the arrays made of silver particles wrapped around a dielectric cylinder, the dipole scattering mode has been canceled^16^. It should be noted that Clausius–Mosotti's effective medium approximation and bi-dimensional model have been used to theoretically demonstrate the functionality of the above meta-surfaces^17^. As further instances, different distributions and orientations of silver ellipsoidal particles have been considered around a dielectric sphere to implement a plasmonic cloak in the visible spectrum^18^. Another scheme for cloaking of the cylindrical objects has been proposed by core–shell nanoparticles around the cylinder and it is proved that perturbation of the periodic alignment does not significantly affect the performance of the structure^19^.

Recently, the capability of the localized surface plasmons of graphene-wrapped spherical nanoparticles in the design of tunable optical meta-surfaces has been investigated^20^. The surface plasmons of a periodic assembly of graphene-coated spherical particles are engineered to design different types of absorbers^21–23^. Following the design procedures of the particle coated optical curved structures, in the present research, a graphene-based optical cover is proposed using spherical nanoparticles to enhance the extinction cross-section of an infinite length dielectric cylinder. The potential of the structure for the double Fano resonance generation is also discussed. In particular, in the plasmonic oligomers, the nanoparticles with optimized sizes, shapes, and inter-particle distances are clustered in a way that the corresponding scattering cross-section contains two peaks (bright modes) having a dip (dark mode) between them, as a result of plasmonic hybridization^24^. Fano resonances can be possibly used for bio-sensing, surface-enhanced Raman scattering (SERS), molecular fluorescence, and solar energy conversion.

In general, the generation of Fano modes can be related to the coupled excitation of various types of resonances. For instance, the destructive interaction between the narrowband and broadband dipolar plasmonic resonances in a gold-graphene sensor results in the plasmonic electromagnetic induced transparency (EIT) with a Fano line-shape^25^. In another approach, a wide dipole resonance is overlapped with narrower higher-order modes spatially and spectrally to give raise to plasmonic induced transparency (PIT)^26^. Moreover, the interface of electric and magnetic dipole modes can easily excite Fano resonances in all-dielectric oligomers^27^. Also, magnetic plasmon modes constructed by circulating currents in the rings of plasmonic nanoparticles lead to the Fano resonances with low radiation loss^28,29^. Another approach is anapole modes which are non-radiating configurations obtained by spectrally overlapped composition of electric and toroidal dipoles^30^. Symmetry breaking is another efficient approach for the Fano resonance generation that can be achieved in various ways including but not limited to the size, position, and incident angle engineering^31–34^. Our proposed device benefits from the incident angle symmetry breaking for the excitation of Fano resonances.

The paper is organized as follows. In “Discussions and results”, a single one-dimensional periodic array of graphene-coated spherical particles is considered around an infinite length dielectric cylinder and the influence of the loci of the particle in the optical performance is investigated. Later, the azimuthal periodicity of the structure is varied by considering a different number of longitudinal one-dimensional periodic arrays around the cylinder. In “The optical performance of the hexamer”, a hexamer is considered as the prototype, and the impact of geometrical, material and optical parameters along with the wave polarization on the extinction cross-section is investigated in detail. Two potential applications of the structure including bio-sensing and optical absorption are illustrated as well. Concluding remarks are mentioned in “Conclusions”.

## Discussions and results

In this section, various combinations of graphene-based plasmonic nanoparticles are considered around a cylindrical dielectric core. Later, the influence of the array position and configuration on the extinction cross-section of the structure is investigated in detail. All the simulations are carried out with the frequency domain solver of the CST 2017 commercial software using the built-in surface conductivity model of graphene material^35^. This model is consistent with the well-known Kubo model of local surface conductivity σ in which graphene is characterized by the relaxation time τ and chemical potential μ_*c*_^36^. Based on this model, graphene is considered as an infinitely thin spherical shell using the convert to sheet option in the software. Note that because of the large number of carbon atoms in the simulated shells, the curvature of the spherical nanoparticles does not affect the surface conductivity model available for the planar sheets^37^. To simulate an infinite length cylinder, image theory is used to set the top, and bottom faces of the solution domain with respect to the cylinder axis to perfect electric conductor (PEC) under TM waves and to perfect magnetic conductor (PMC) for TE waves. The details of image theory can be found in classic electromagnetic books^38^.

### Cylindrically-wrapped graphene-based plasmonic oligomers

**Figure 1:**
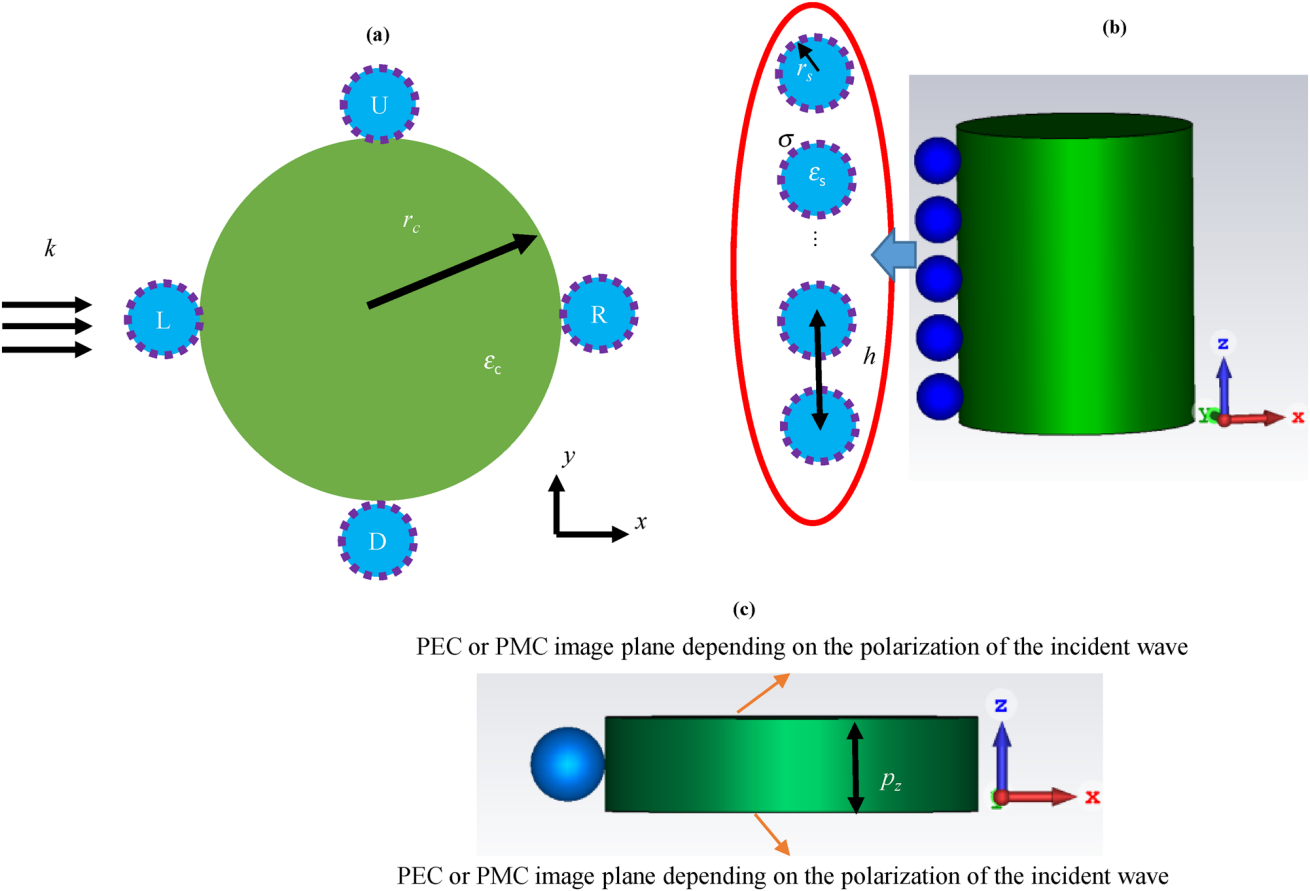
A longitudinally infinite one-dimensional array constructed by the graphene-coated hollow spherical nanoparticles wrapped around a dielectric cylinder, on the possible positions of R (Right), L (Left), U (Up), or D (Down) with respect to the incident wave (**a**) 2D and (**b**) 3D views. When the nanoparticles located at two of the labeled positions are considered simultaneously the double one-dimensional periodic array is attained. (**c**) The 3D view of the simulated unit cell for the L position. In simulations, the periodicity along the cylinder axis is implemented via the image theory.

The structure under consideration is a dielectric cylinder with the radius *r*_*c*_ = 500 nm and core permittivity ε_c_ = 2 under plane wave illumination, as shown in Fig. 1. It is considered that the cylinder axis resides along the *z*-axis and the linearly polarized wave is transverse electric (TE) with respect to it. It is also assumed that a single one-dimensional periodic array with the periodicity along the cylinder axis covers the wire. The array is constructed by graphene-coated hollow spherical nanoparticles with the longitudinal periodicity of *h* = 250 nm. The possible locations of the array with respect to the plane wave are denoted by L (Left), R (Right), U (Up), or D (Down). The initial parameters are as follows: the radii of the spheres are *r*_*s*_ = 100 nm, relaxation times of graphene shells are τ = 1.5 ps, and chemical potentials of graphene shells are μ_*c*_ = 0.5 eV.

**Figure 2:**
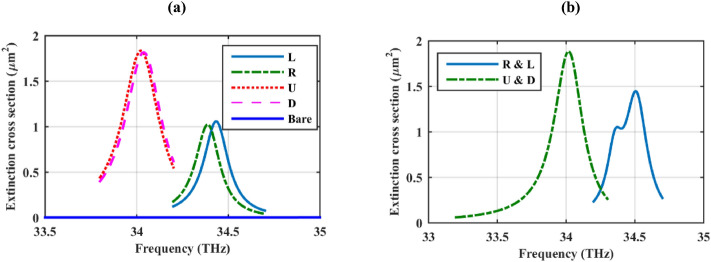
The extinction cross-section of the structure in Fig. 1 and its comparison with the bare cylinder. In (**a**) only one and in (**b**) double one-dimensional periodic arrays of nanoparticles are considered at the specified locations in Fig. 1. The design parameters are as follows: *r*_*c*_ = 500 nm, ε_c_ = 2, *r*_*s*_ = 100 nm, *h* = 250 nm τ = 1.5 ps, and μ_*c*_ = 0.5 eV. The spherical particles are considered hollow.

Initially, a one-dimensional periodic array is considered in R, L, U, or D locations concerning the incident wave, as illustrated in Fig. 1. Figure 2a shows the extinction cross-section of this structure and its comparison with the bare cylinder. The extinction cross-section is attained by summing the scattering and absorption cross-sections^39^ which are calculated using broadband far-field monitors in the simulations. It is observed that the presence of a one-dimensional periodic array of plasmonic nanoparticles enhances the extinction cross-section of the dielectric cylinder, considerably. This effect is due to the excitation of localized surface plasmons of the graphene shells and it depends on the position of the array with respect to the incident wave, which alters the incident angle of the illuminating wave. Therefore, as Fig. 2b confirms, by considering double one-dimensional periodic arrays of the labeled particles in the simulation, the extinction cross-section can be further manipulated by considering the arrays in different positions with respect to the incident wave.

**Figure 3:**
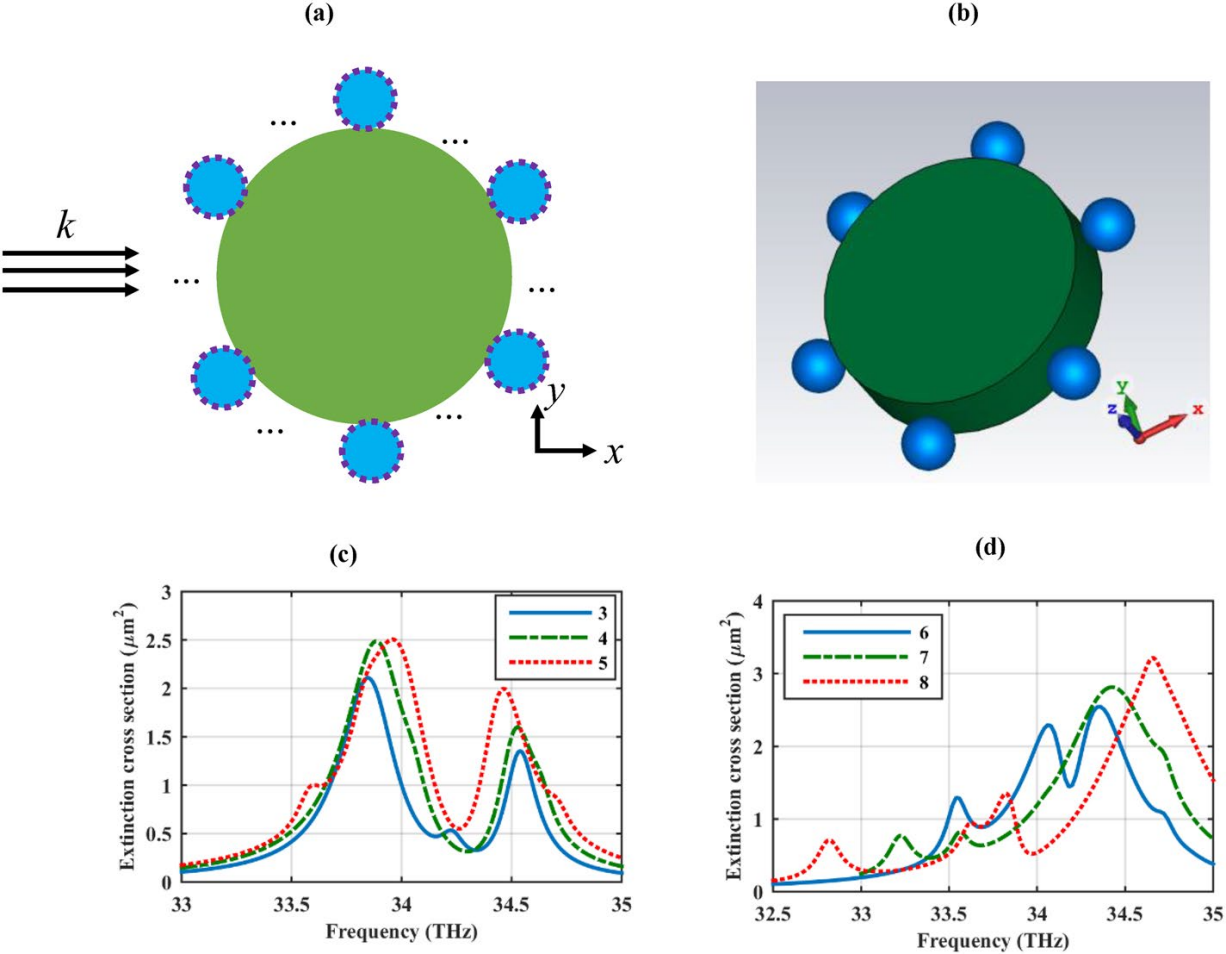
An array of polarizable nanoparticles with a different number of one-dimensional periodic arrays, arranged around an infinite length dielectric cylinder (**a**) 2D and (**b**) 3D views. Dashed lines represent graphene coating. The extinction cross-section considering various numbers of one-dimensional periodic arrays around the cylinder (**c**) 3–5 and (**d**) 6–8. The design parameters are as follows: *r*_*c*_ = 500 nm, ε_c_ = 2, *r*_*s*_ = 100 nm, *h* = 250 nm, τ = 1.5 ps, and μ_*c*_ = 0.5 eV. The spherical particles are considered hollow. Note that in the 3D view, only a unit cell is illustrated and the periodicity along the cylinder axis is implemented via the image theory in the simulations.

The impact of increasing the number of one-dimensional periodic arrays around the cylinder on its extinction cross-section is investigated in Fig. 3. Specifically, 3–8 one-dimensional periodic arrays are considered around the dielectric cylinder as illustrated in Fig. 3a,b schematically. This modification alters the azimuthal periodicity of the particles. The resulted extinction cross-sections are illustrated in Fig. 3c,d and it is understood that by tailoring the number of one-dimensional periodic arrays the mutual interaction of the excited localized surface plasmons results in the increased extinction cross-section. Moreover, for each nanoparticle array, there is at least a Fano dip in the extinction curve. The origin of the Fano resonance can be understood by considering the structure as an oligomer with 1D periodicity rather than the conventional planar ones. Specifically, nanoparticle oligomers in the form of the dimer, trimer, quadrumer, pentamer, heptamer, and higher-order assemblies are some of the most promising ways to produce Fano resonances^40^. These structures, mainly have 2D periodicity which is attained by pattering the oligomer on the planar surface^28,41–44^. The generation of the Fano resonances in our proposed structure benefits from the same strategy using a cylindrical substrate. Symmetry breaking provided by the different incident angles that each particle experience is the main mechanism for the Fano resonance generation. Note that for hexamer, heptamer, and octamer configurations, there are double Fano dips in the extinction cross-section. The meta-molecules can be engineered for the modification of the plasmon line-shape in double spectral positions, simultaneously, which have great potential in dual plasmonic sensor applications^45^. In this regard, a metallic double Fano-resonant nano-cluster is proposed as a four-wave mixing device^46^ and our proposed structure presents a novel double Fano resonant structure using graphene plasmons.

Note that a well-known approach for the extinction (scattering) cross-section enhancement of the cylindrical objects is exploiting the super-scattering phenomenon^2,3,9,10^. The main drawbacks of this method are its extreme sensitivity to the fabrication tolerance and material losses due to its highly resonant behavior. Therefore, very limited realizations of them are available in the low-frequency window^11^. Our proposed device performs more robustly considering these two aspects, as will be further clarified by the parametric analysis of the performance. Moreover, the invisible cloak design using plasmonic particle covers with different morphologies is a well-established topic^15–19^. Interestingly, the extinction cross-section enhancement can be considered as the opposite side of cloaking, where sensing is of interest rather than hiding. This purpose is attained using the same geometry as the previously published ones for the cloaking. The aforementioned cloaks can be fabricated by the self-assembly technique and have a robust performance against fabrication imperfections^19^. Since the graphene-coated hollow particles can be realized chemically^47^, the proposed cover can also be fabricated with the self-assembly technique.

### The optical performance of the hexamer

To further investigate the performance of the cylindrical wire with a cover constructed by graphene-coated particles, the hexamer configuration is considered as a prototype, and the impact of various material, optical, and geometrical parameters on the extinction cross-section of the wire is investigated. Before proceeding to the next sections, the excited localized surface plasmons on the three peaks of the extinction cross-section of the hexamer (Fig. 3b) are illustrated in Table 1. The second-order resonances with different degrees are observed^23^. These resonances are the main mechanism of the performance.

**Table 1:**
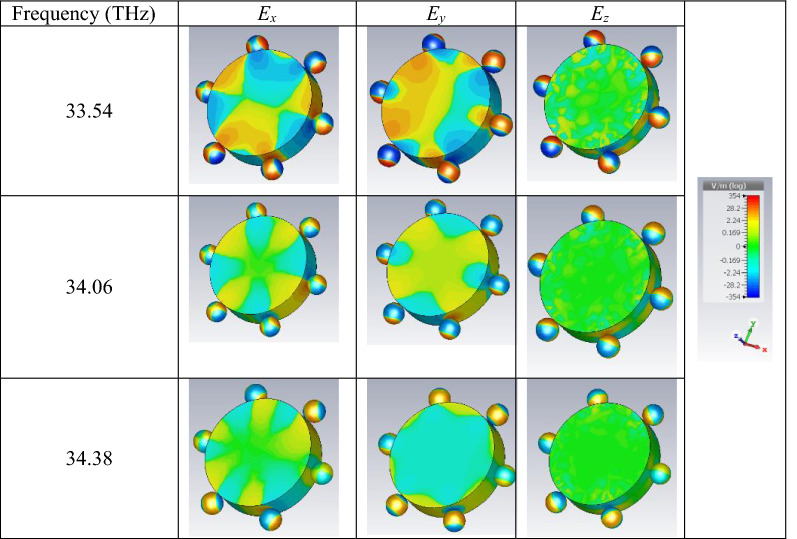
Excited localized surface plasmons at the extinction peaks of the hexamer (Fig. 3b).

### Material parameters

**Figure 4:**
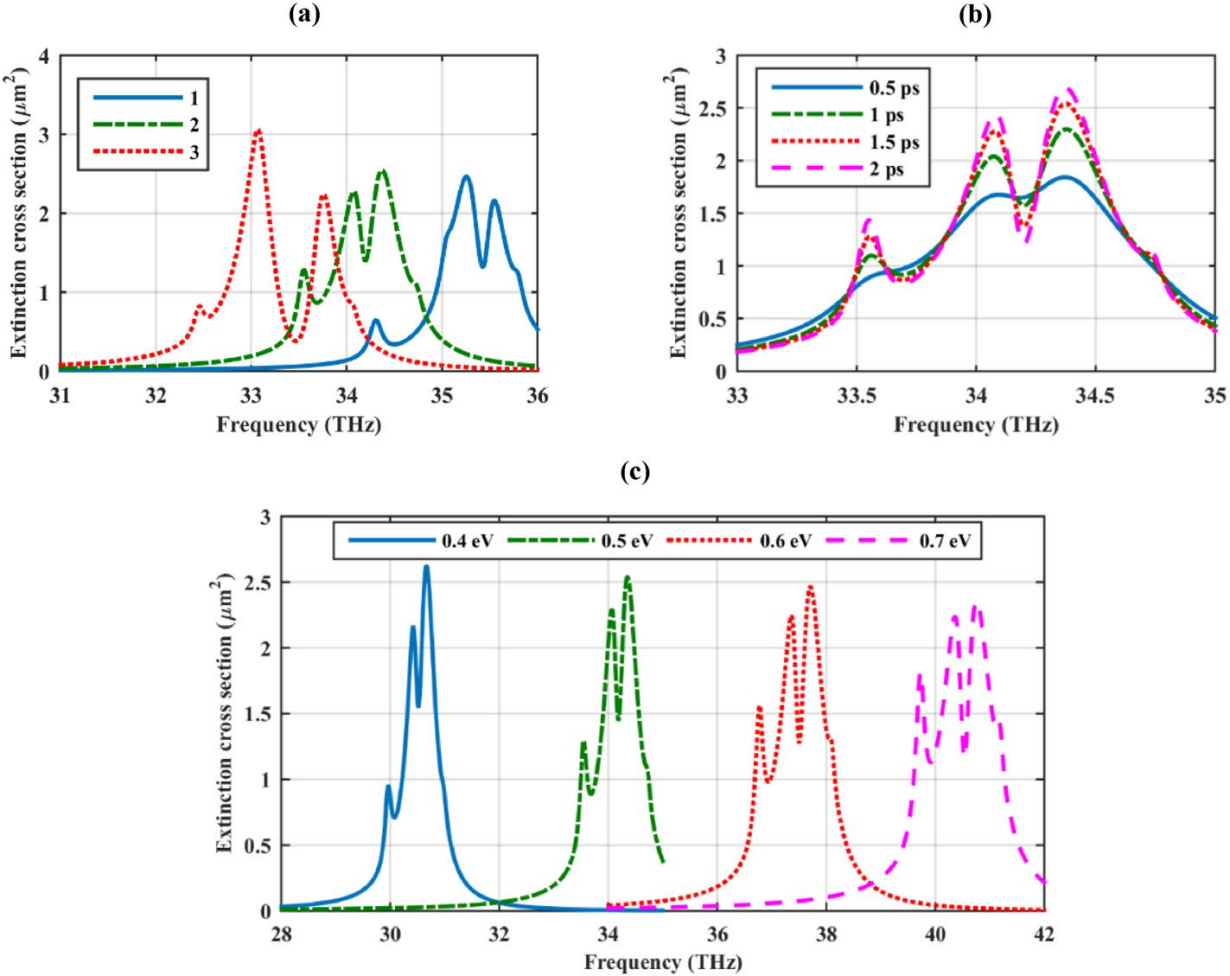
Variations of the extinction cross-section of the dielectric cylinder with hexamer cover by considering different (**a**) dielectric cores (**b**) graphene relaxation times and (**c**) graphene chemical potentials. The initial design parameters are as follows: *r*_*c*_ = 500 nm, ε_c_ = 2, *r*_*s*_ = 100 nm, *h* = 250 nm, τ = 1.5 ps, and μ_*c*_ = 0.5 eV. The spherical particles are considered hollow.

This subsection studies the influence of the core material and graphene optical parameters on the extinction cross-section of the hexamer. As Fig. 4a shows, there are two Fano dips for each considered dielectric core. Moreover, by increasing the dielectric constant of the core, the high-frequency Fano dip experiences a red shift and becomes more pronounced. The low-frequency Fano dip gradually disappears by increasing the core permittivity. In the next simulations, optical properties of graphene material are manipulated in the experimentally realizable ranges, which is up to 3 ps for the relaxation time and up to 2 eV for chemical potential^48^. Based on Fig. 4b, the relaxation time of the graphene shells modulates the Fano dips without altering their spectral positions. High-quality graphene materials are suitable for reaching deeper Fano resonances. This observation is consistent with the scattering analysis of graphene-coated nanoparticles^49,50^. The proposed sub-wavelength plasmonic cover can be simply reconfigured by changing the bias voltage/doping density of the graphene covers. This fact is illustrated in Fig. 4c by varying the chemical potential from μ_*c*_ = 0.4–0.7 eV. As the figure shows, by increasing the chemical potential, the resonance frequency experiences a blue shift. This feature is unattainable with metal-based plasmonic oligomers and has only been implemented using a planar assembly of graphene-based disk-shaped particles^34,51^.

**Figure 5:**
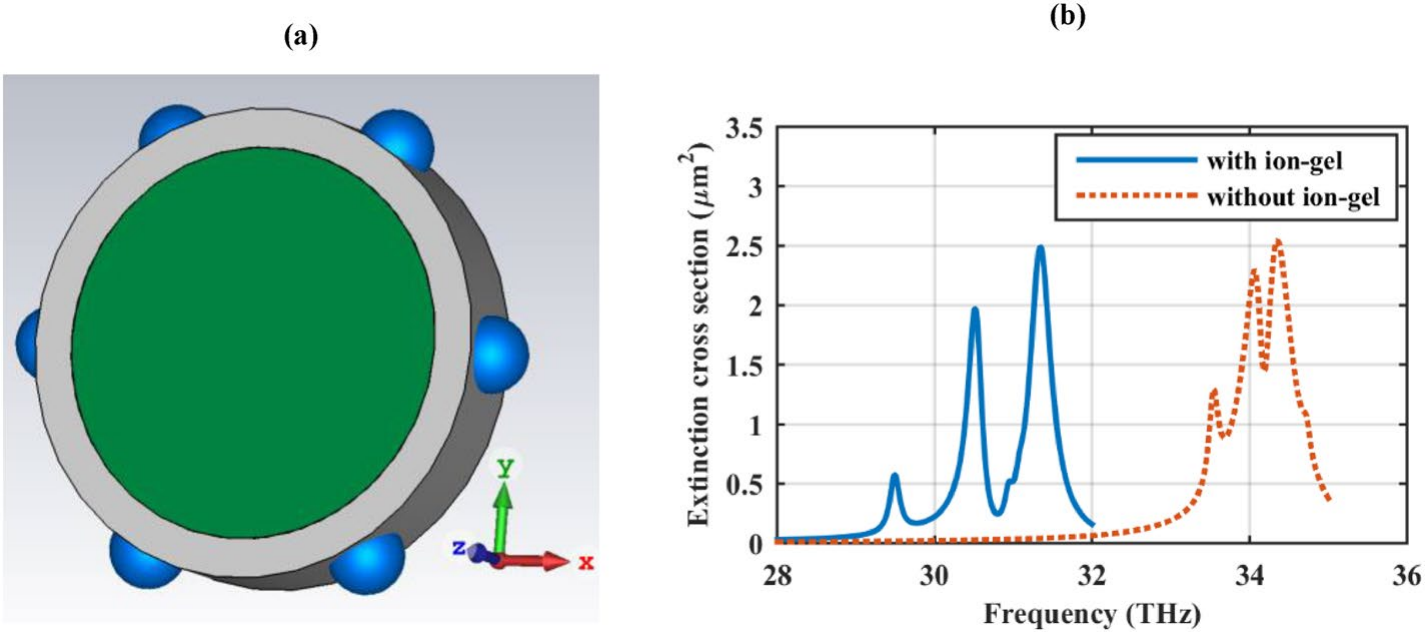
(**a**) The 3D geometry of the dielectric cylinder with hexamer cover biased by an ion-gel layer (gray color) and (**b**) its extinction cross-section. The design parameters are as follows: *r*_*c*_ = 500 nm, ε_c_ = 2, *r*_*s*_ = 100 nm, *h* = 250 nm, τ = 1.5 ps, and μ_*c*_ = 0.5 eV. The spherical particles are considered hollow. The thickness and refractive index of the ion-gel layer are respectively 100 nm and 1.43.

Modulating the chemical potential of graphene shells is an important issue in practice. A simple approach for this purpose is to electrically connect the particles using cross-hair geometry or reside them on top of another simply biased structure^23,52^. In order to obviate the need for further modification of the geometry, the ion-gel method is proposed as another approach, which has been widely used for planar structures^53–55^. As Fig. 5a illustrates, a 100 nm thick ion-gel layer with the refractive index of *n*_gel_ = 1.43 covers the particles^54^. The resulted extinction cross-section and its comparison with the initial design are provided in Fig. 5b, confirming the presence of the previously mentioned phenomenon in different spectral positions. Note that the geometry can be further modified by inserting a metal core for the practicability^7^.

### Geometrical parameters

**Figure 6:**
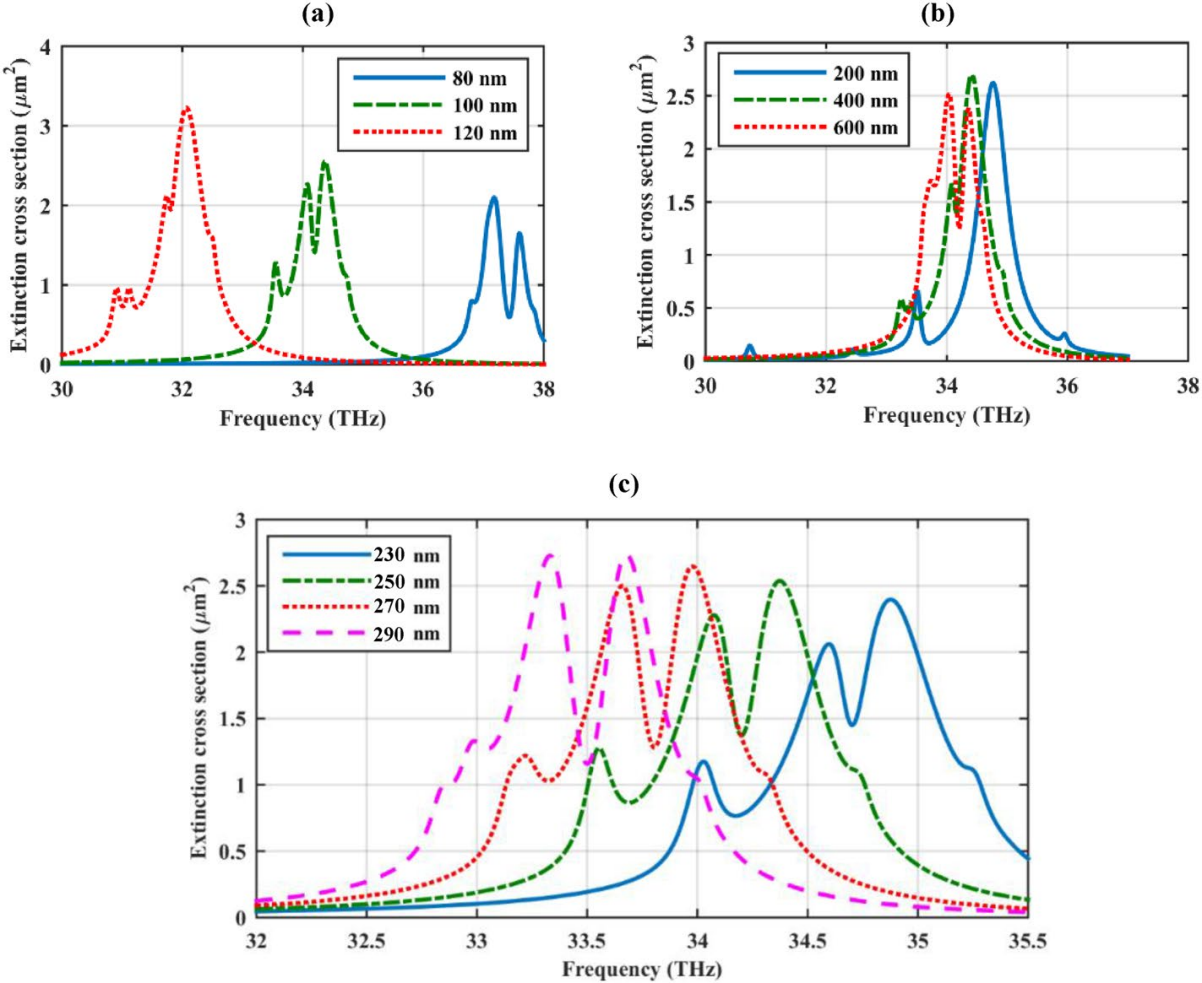
The impact of the geometrical parameters on the modulation of the Fano dips (**a**) the radii of the spheres (*r*_*s*_) (**b**) the radius of the cylinder (*r*_*c*_) and (**c**) the periodicity along the cylinder axis. The initial design parameters are as follows: *r*_*c*_ = 500 nm, ε_c_ = 2, *r*_*s*_ = 100 nm, *h* = 250 nm, τ = 1.5 ps, and μ_*c*_ = 0.5 eV. The spherical particles are considered to be hollow.

The impact of the geometrical parameters on the modulation of the Fano dips is illustrated in Fig. 6. Based on Fig. 6a, the Fano dips have a large nonlinear sensitivity to the radii of the particles. The second Fano dip is more pronounced for spherical particles with smaller radii and it occurs at higher frequencies following the plasmonic behavior of graphene-coated particles with different radii^37^. Note that the longitudinal periodicity is fixed in all the previous simulations. Moreover, cylinders with larger core radii exhibit a remarkable Fano dip at high frequencies, as illustrated in Fig. 6b. Finally, based on Fig. 6c, by increasing the longitudinal periodicity, the first Fano resonance has gradually disappeared while the second Fano resonance becomes deeper and happening at lower frequencies. From the two latter discussions, it can be inferred that the first Fano resonance is highly affected by the plasmonic coupling of the particles.

### Polarization of the incident wave

**Figure 7:**
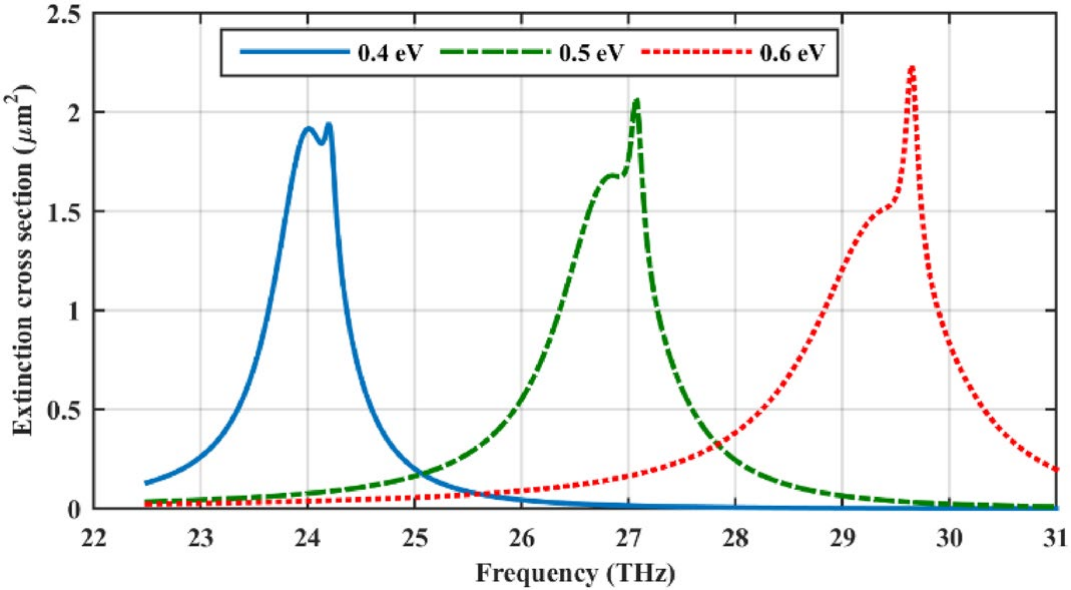
Impact of the polarization of the incident wave on the extinction cross-section of the dielectric cylinder coated with six one-dimensional periodic arrays. TM polarized plane wave is considered in this case. The initial design parameters are as follows: *r*_*c*_ = 500 nm, ε_c_ = 2, *r*_*s*_ = 100 nm, *h* = 250 nm, τ = 1.5 ps, and μ_*c*_ = 0.5 eV. The spherical particles are considered hollow.

An outstanding feature of our proposed cylindrical structure is its dual-polarized optical performance. To discuss this feature, in Fig. 7, the polarization of the incident electromagnetic field is considered as TM. As the figure indicates, the extinction cross-section of the cylinder is considerably enhanced in comparison to the bare cylinder. Also, the extinction resonance frequency can be modulated considering different chemical potentials for the graphene shells. Using a continuous graphene shell, the structure performs only under TE polarization^56,57^ and this outstanding feature is attained thanks to using a spherically symmetric cell.

### Applications

**Figure 8:**
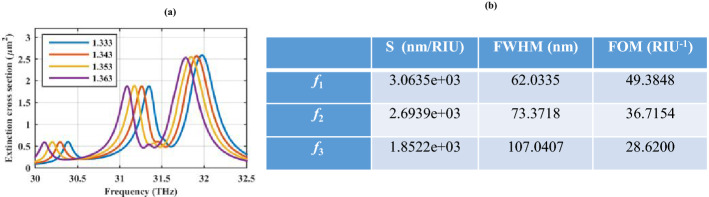
(**a**) Investigating the refractive index sensing capability of the hexamer cover by changing the refractive index of the gray layer in Fig. 5a from *n* = 1.333 to 1.363 and (**b**) the sensor parameters. The initial design parameters are as follows: *r*_*c*_ = 500 nm, ε_c_ = 2, *r*_*s*_ = 100 nm, *h* = 250 nm τ = 1.5 ps, and μ_*c*_ = 0.5 eV. The spherical particles are considered hollow. *f*_1_–*f*_3_ respectively denote the first to the third resonance frequencies.

Let us reconsider the structure in Fig. 5a and replace the ion-gel material with an analyte layer with the refractive index in the range of *n* = 1.333–1.363 to investigate the sensing performance of the generated Fano resonances. The extinction cross-section of the structure is illustrated in Fig. 8a and the evaluation of the sensing performance using sensitivity (S), full width at half maximum (FWHM), and figure of merit (FOM) parameters^58^ are included in Fig. 8. In comparison to the planar Fano resonance-based plasmonic heptamer nanohole array sensor with the highest sensitivity of ~ 400 nm/RIU and the highest figure of merit of ~ 24 RIU^−1^^59^, our proposed structure performs better in all three plasmonic resonances due to the excitation of higher order modes (shown in Table 1).

**Figure 9:**
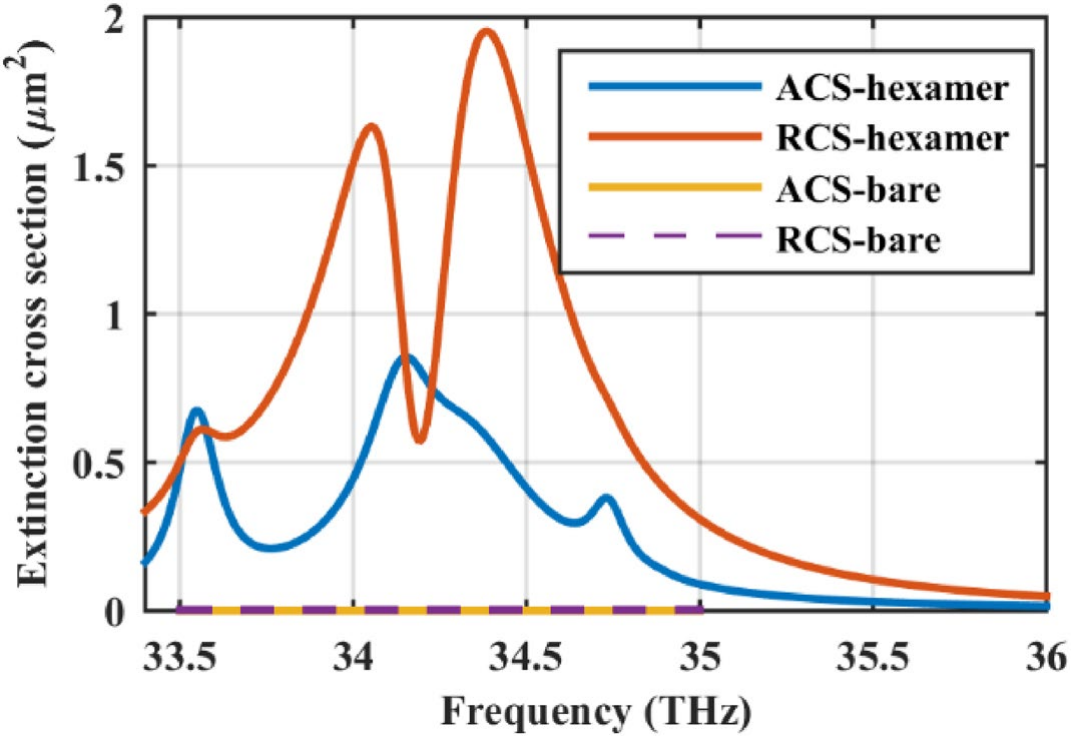
Comparison of the absorption cross-section (ACS) and scattering cross-section (RCS) of the hexamer with the bare cylinder. The initial design parameters are as follows: *r*_*c*_ = 500 nm, ε_c_ = 2, *r*_*s*_ = 100 nm, *h* = 250 nm, τ = 1.5 ps, and μ_*c*_ = 0.5 eV. The spherical particles are considered hollow.

As a final note, the absorption cross-section (ACS) and scattering cross-section (RCS) of the hexamer are compared with the bare dielectric wire in Fig. 9 to confirm the potential of the proposed structure in the absorber design.

## Conclusions

One-dimensional and two-dimensional arrays of polarizable nanoparticles constructed by graphene-coated spherical particles have the capability of enhancing the extinction cross-section of a dielectric cylinder. By adjusting the number of particles, the amount of the extinction cross-section can be tuned. The structure has many degrees of freedom for optical response manipulation and it performs for both TE and TM polarizations. Our proposed structure can be possibly used is in the design of sub-wavelength reconfigurable absorbers for sensing applications.
